# Clinical Implications for the Comprehensive Interpretation of Radiologic and Immunodiagnostic Tests in Patients Suspected of Parasitic Hepatic Cyst, a Rare Case in Korea

**DOI:** 10.3390/tropicalmed8030155

**Published:** 2023-03-02

**Authors:** Jae-Sung Yoo, Min-Kyu Kang, Jung-Gil Park, Hyung-Joo Kim, Joon-Hyuk Choi

**Affiliations:** 1 Department of Internal Medicine, Yeungnam University Medical Center, Daegu 42415, Republic of Korea; 2 Department of Internal Medicine, College of Medicine, Yeungnam University, Daegu 42415, Republic of Korea; 3 Departments of Surgery, Yeungnam University Medical Center, Daegu 42415, Republic of Korea; 4 Department of Pathology, College of Medicine, Yeungnam University, Daegu 42415, Republic of Korea

**Keywords:** cystic echinococcosis, hydatid disease, neglected tropical disease

## Abstract

Cystic echinococcosis (CE) is a representative neglected tropical disease (NTD) with increased morbidity and mortality but is ignored and overlooked in developed countries. Serological and radiographic findings are helpful in distinguishing these parasites; however, conflicting results of these can make it difficult to diagnose if medical knowledge of hepatic parasitic disease, including the etiology, features of imaging, and immunodiagnostic test, is not acquired. We report the case of a male patient with dyspepsia and right epigastric pain who had positive results for cysticercosis antibodies on immunodiagnostic examination. Abdominal ultrasonography revealed two huge communicating cystic lesions measuring 8–11 cm. Further evaluations for cysticercosis of the brain (neurocysticercosis) and eyes (intraocular cysticercosis) were unremarkable throughout the brain imaging test and fundus examination. A laparoscopic right hemi-hepatectomy was performed for diagnosis and treatment. On histopathological examination, diverse stages of *Echinococcus granulosus* were identified. Albendazole was administered postoperatively, and the patient was also followed up. We should be aware of the etiologies that have been prevalent in parasite infection thought to be the cause of hepatic cysts. Moreover, we make an effort to ascertain the patient’s nationality, past travel experiences, and immediate environment, including any animals and pets. We present the case of a patient who was worried about the possibility of liver invasion of cysticercus due to the positivity of the cysticercosis antibody and was ultimately diagnosed with CE.

## 1. Introduction

Hepatic cysts are often seen in clinical practice and could possibly have a variety of characteristics including being benign, pre-malignancy, malignancy, traumatic, and infectious condition. The infectious hepatic cyst was further divided into parasitic and non-parasitic, and the most common disease among hepatic parasitic cysts is cystic echinococcosis (CE) [1]. CE, which is a neglected tropical disease (NTD), is a zoonotic disease that is caused by parasites. This disease classified as an NTD shares two significant similarities. First, this illness is more prevalent in warm climate regions than in cool climate ones. However, this propensity for warmer regions is mostly due to the distribution of poverty in desperate refugees, urban slums, and isolated rural settlements that are close to the equator. In developed countries, this disease was overlooked and ignored. Second, NTDs tend to be neglected by funders, policymakers, and researchers [2]. Due to this characteristic of NTDs, they are a socio-economic burden and cause severe morbidities and mortalities [3]. CE, also known as hydatid disease, is caused by the larval form of the tapeworm *Echinococcus granulosus*. This disease has two major diagnostic tools: radiological imaging modalities and serum immunodiagnostic tests [4]. CE is usually diagnosed using ultrasonography (USG) and computed tomography (CT). Among these, USG is the most common diagnostic tool. Unlike radiologic modalities, serologic tests are inconclusive and have difficulty in differential diagnosis with other parasitic infections due to cross-reactivity [5]. Due to this limitation, there is ambiguity in the initial definite diagnosis. Herein, we will report a case in which a patient who was mindful of the possibility of hepatic invasion of cysticercosis due to a positive cysticercosis result was finally diagnosed with CE. Through this case, we would like to discuss the comprehensive interpretation of various diagnostic results suggesting hepatic parasite infection, a rare case in South Korea.

## 2. Case Presentation

**Figure 1:**
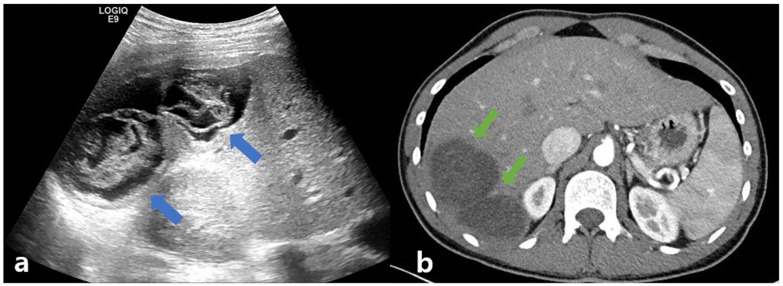
Abdominal ultrasonography (**a**): two communicating, incomplete loculated anechoic lesions, measuring approximately 8–11 cm, filled with folded linear echoic materials (blue arrows). Abdominal computed tomography (**b**): two communicating hypodense cystic lesions, measuring approximately 8–11 cm, filled with slightly enhancing material (green arrow).

A 23-year-old man, who presented with dyspepsia and intermittent right upper quadrant pain for several weeks, was transferred to a tertiary hospital because of the abnormal findings on CT, including a septal low-density lesion. He was 71.8 kg in weight, 181cm tall, and had a body mass index of 21.92. He had no previous history of taking medications or alcohol and no relevant medical history. He was a foreign worker from Uzbekistan who had migrated 3 years prior, and he was not trying to recall if he had ever interacted with livestock or pets. He claimed to have lived in the urban city and was a student while in Uzbekistan. Currently, he is working at mechanical parts factories, less relevant for parasites. For further evaluation, laboratory examinations, including liver function tests, parasite analysis, and hepatobiliary sonography, were performed. The results of the laboratory tests were as follows: white blood cell (WBC) count, 6560/μL; eosinophilia, 860 cells/μL (proportion of WBC, 13%); hemoglobin, 15 g/μL; platelet count, 200 K/μL; total protein, 7.82 g/dL; albumin, 4.47 g/dL; total bilirubin, 0.62 mg/dL; direct bilirubin, 0.23 mg/dL; aspartate aminotransferase (AST), 20 IU/L; alanine aminotransferase (ALT), 19 IU/L; alkaline phosphatase, 104 IU/L; gamma glutamyl transferase, 36 IU/L; blood urea nitrogen, 12.3 mg/dL; creatinine, 0.87 mg/dL; and international normalized ratio, 1.12. Serologic markers for viral hepatitis, including the hepatitis B virus surface antigen, hepatitis B virus core antibody, hepatitis B virus antigen, and hepatitis C virus antibody, were negative. The hepatitis B virus surface antibody was positive. The laboratory test for autoimmune hepatitis, such as the anti-nuclear antibody, smooth muscle antibody, liver kidney microsomal type 1 antibody, and antimitochondrial antibody, were also negative. Additional laboratory findings for parasites, including serum Toxocariasis, *C. Sinensis*, Cysticercosis, *Paragonimus*, *Fasciola hepatica*, and *Sparganum* immunoglobulin G (IgG), were performed. Of these, only the cysticercosis IgG test was positive. Abdominal ultrasonography showed two communicating, incomplete loculated anechoic lesions measuring approximately 8–11 cm and filled with folded linear echoic materials in liver segment 6. (Figure 1a) Abdominal computed tomography revealed two communicating hypodense cysts occupying a slightly enhancing material. (Figure 1b).

**Figure 2:**
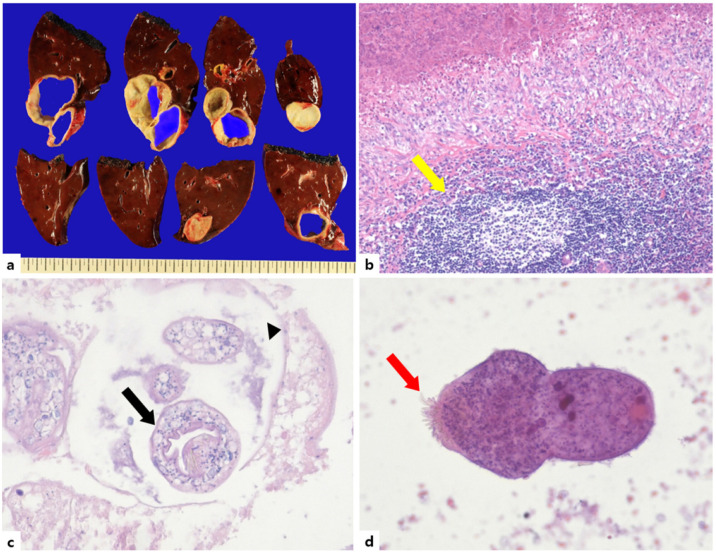
Gross appearance (**a**) Histopathology findings (**b**–**d**). (**a**) Gross finding. The huge hepatic cyst separated by multiple lobules. (**b**) A fibrinous wall and an infiltration of inflammatory cells, including lymphocytes, eosinophils, and histiocytes, are seen in the liver. (Yellow arrow, H&E ×100). (**c**) Protoscolices (Black arrow) and brood capsule (Black arrowhead) are noted (H&E ×200). (**d**) Microscopic examination of the pathological specimen demonstrating the scolex with visible hooklets (Red arrow) (H&E ×200).

Based on the results of the serologic and radiologic examinations, we initially suspected parasitic infection, but a specific organism could not be confirmed. So, the possibility of liver involvement in cysticercosis could not be ruled out. We consulted the neurosurgery and ophthalmology departments to check for cysticercosis in other organs; however, no lesions were found in the brain and eyes throughout the brain imaging test and fundus examination. After multidisciplinary discussion, we decided to proceed with surgical intervention. Given its size, a laparoscopic right hemi-hepatectomy was performed. The gross weight of the specimen was 1096.5 g, and it measured 21.5 × 14.0 × 6.2 cm; however, the size of the cyst was 10.0 × 9.5 × 5.5 cm, and it was a multi-loculated cyst. Histopathological examination revealed multiple granulomas caused by *Echinococcus*, which were composed of lymphocytes, eosinophils, and histiocytes infiltrating the liver’s fibrinous wall. Several daughter cysts composed of brood capsules containing protoscoleces were observed. Microscopic examination of a hematoxylin and eosin (H&E) stain at 200× magnification showed a scolex with visible hooklets. (Figure 2) On histopathology, *Echinococcus* spp. was confirmed, and the final diagnosis was CE. No complications occurred during or after surgery, and an administration of 1000 mg of albendazole (ABZ) was initiated based on a body weight of approximately 70 kg (10–15 mg/kg). The patient will be followed up in an outpatient department.

## 3. Discussion

CE is frequently reported worldwide, particularly in northern Russia, Siberia, China, Central Asia, other parts of Africa, and South America [6,7,8]. Particularly, the country of the patient was Uzbekistan in our case. Since the collapse of the Union of Soviet Socialist Republics in 1991, Central Asia, which includes Kazakhstan, Uzbekistan, Kyrgyzstan, Tadjikistan, and Turkmenistan, underwent changes in their social and economic environments. Due to the lack of budget to maintain the system and manpower supervising the slaughters, the reorganization of the livestock industry proceeded from a large scale of automated slaughterhouses to small farms. Along with this tendency of transformation, new issues arose. First, unsupervised home slaughtering and market slaughtering without veterinary quality control have become more common. Second, the population of dogs increased as the demand for pets and guard dogs increased. A comparable rise in CE has also occurred. According to official statistics, 387 surgical cases of echinococcosis were documented in 1988. By 1998, this had grown to 1438 cases, 5.8 cases per 100,000 people, which is 3.7 times more. In 2000, there were 1435 cases, and in 2001, there were 819 cases. The number of CE cases in 2010 was 2966 patients. However, these numbers of CE cases were underestimated. This is due to the fact that the data only included a minority of the emergency department or other hospital patients who were identified at the local downtown medical centers. Due to the lack of a cooperation structure between hospitals and the state Sanitation and Epidemiology Centers, many of them were ignored [9]. Recently, the update of the surgical incidence of CE from 2011 to 2018 was analyzed. To overcome previously mentioned limitations, the surgically treated CE cases in licensed institutions were gathered nationally. There were 7309 CE cases documented from 2011 to 2018. The incidence rates ranged from 4.4 per 100,000 people in 2011 to 2.3 per 100,000 people in 2018. Grossly, it was statistically significant to observe a decline in most areas [10]. However, the cases of CE were rare in Korea. Since the first report of echinococcosis in 1983, about 38 cases were reported by 2019. Among them, the number of hepatic involvement of echinococcosis was 22 cases [11]. As recently reported, 10 cases of CE were reviewed, 4 of 10 patients were Uzbekistans, and the others were Koreans, one of whom had a travel history to Uzbekistans [12].

There are three forms of echinococcosis: human CE, alveolar echinococcosis, and polycystic echinococcosis, caused by *E. granulosus*, *E. multilocularis*, and *E. vogeli*/*Echinococcus oligarthus*, respectively [13]. The most prevalent form of the disease, accounting for more than 95% of the estimated 2–3 million cases worldwide, is human CE [14]. In the life cycle of *E. granulosus*, the adult worm is mainly located in the small intestine of carnivores such as dogs, which are definitive hosts [14]. The intermediated hosts, including herbivores, were infected by feces of the definitive host, which contain the larval phase of *E. granulosus*. Human beings are aberrant intermediate hosts that accidentally ingest embryonated eggs, which emerge as metacestodes (larvae) in the liver after hatching in the intestine and releasing oncospheres that migrate through the portal and lymphatic spreading [15]. In our case, the patient did not recall any history of contact with livestock or pets, but he may have had some experience with it.

The hydatid cyst formation was initiated from two portions, the echinococcal organism (or hydatid) and adventitia. The host’s organ response to the hydatid, a foreign body, wraps up in the adventitia, a layer of quiescent tissue with fibrosis and diverse thickness. Consequently, the layer of the hydatid cyst was made up of three layers, including the geminal layer, laminated layer, and adventitia layer [16]. Among them, the hydatid is subdivided by two layers, the laminated layer and geminal layer. The germinal layer consists of embryonic cells. Through reproduction processes that guarantee a fertility cyst, the germinal layer produces brood capsules, which contain protoscoleces and scolices. The brood capsules containing protoscoleces invaginated in an acellular laminated layer make up the various-sized daughter cysts.

The protoscoleces could pursue two direction of life strategies. One grows into an adult tapeworm that lays sexually reproduced eggs in the dog’s gastrointestinal systems. In the other, each released protoscolex can differentiate asexually into a new cyst if a hydatid cyst ruptures within the intermediate or human host; this is known as secondary echinococcosis [15].

According to the difference in the way the disease affects, echinococcus is classified as primary or secondary echinococcosis. In the human being, cysts may emerge in a variety of organs after the oral route of *E. granulosus* eggs. This type of echinococcosis is referred to as primary CE. Secondary CE, which mostly affects the abdominal cavity, is caused by a cyst rupture led on by trauma or spontaneously, releasing protoscoleces and/or tiny daughter cysts, which may develop into huge cyst.

The hydatid cyst is filled with liquid material containing the secretion of the echinococcal organism and host. Due to this composition of the cyst, it has antigenic traits that provoke an immune reaction and anaphylaxis.

In the present case, we can observe a diverse transmitted phase of *E. granulosus*. Protoscolex embedded in daughter cysts and roaming scolex could be observed.

**Figure 3:**
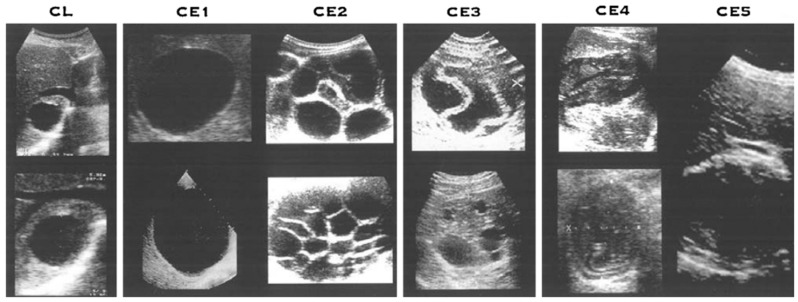
WHO-IWGE (World Health Organization Informal Working Group on Echinococcosis) criteria standardized classification for cystic echinococcosis [23]. CE, cystic echinococcosis.

The most common organ for cyst development is the liver (65–70%), followed by the lung (20–25%) [13,16,17,18,19]. In these cases, the multiple cysts had variable sizes and shapes according to their stages [20,21]. In echinococcosis, CE was categorized by the WHO-IWGE (World Health Organization Informal Working Group on Echinococcosis) in 2003 and revised for diagnosis and management in 2010 [22,23]. It was classified into CE1 and CE2 as the active phases, CE3 as the transition phase, and CE4 and CE5 as the inactive phases. CE1 was shown as a single, double-lined anechoic cystic lesion, and CE2 was shown as a multi-separated cystic lesion, which was called a “rosette-like” or “honeycomb” cyst. CE3 was further differentiated into CE3a, which is characterized by detachable cysts (lily sign), and CE3b, which is characterized by daughter cysts on a solid substrate. CE4 was a cystic lesion that was filled with heterogenous materials (hyper-/hypo-echoic) without daughter cysts, and CE5 was a cyst surrounded by a calcified wall. (Figure 3) The treatment options should take into account the cyst type, size, and location and the presence of complications. Treatment options for CE include anti-helminth (ABZ) and non-surgical intervention (e.g., puncture, aspiration, injection of a scolecidal agent, re-aspiration (PAIR)), surgery, and a “watch-and-wait” approach). For determining the CE treatment options, the WHO-IWGE US classification, size, location, presence or absence of complications, and the availability of medical competence and equipment were taken into consideration.

In principle, for curative treatment, CE must be completely removed. If all layers of the cyst, including the adventitia, have not been removed (e.g., subtotal cystectomy and PAIR), the protoscolecidal agents should be used. The main causes of CE recurrence include intraoperative protoscolex-rich fluid spread after surgery and inappropriate protoscolece and germinal membrane rupture during percutaneous interventions [23].

In CE1 and CE3a, if the size of the cyst is less than 5 cm, ABZ administration alone may be sufficient, but in cases of 5 cm or more, transdermal PAIR and ABZ administration are preferred. In CE2 and CE3b, catheterization and surgical removal was recommended in addition to anti-helminth. The “modified catheterization technique” (MoCAT), an intervention suitable for cysts up to 10 cm in diameter that aspirates the cyst fluid through the parasite membranes and keeps a catheter in place for the duration of the postintervention period, is a recent advancement in PAIR. This method, when used by skilled professionals, can be an alternative to surgery for uncomplicated CE2 and CE3b cysts. In the case of CE4 and CE5, a “watch-and-wait” strategy may be appropriate.

According to the ultrasound results, the patient in this case was classified into CE3a. Based on the proposed size criteria, this patient was classified as having a large cyst (>10 cm). An additional consideration is rupture risk. Rupture is a severe complication that can induce anaphylaxis [24,25]. The risk factors for rupture are young age, superficial localization, trauma, and large size [26]. Since our patient was young and had a large cyst, surgery was performed. After surgery, the final diagnosis was confirmed to be CE.

When the cyst has been obliterated, administering ABZ from one week before to two months after the interventional treatment (surgery or PAIR) is a recommended prescription based on pharmacological data and the relatively low and slow effectiveness of ABZ to eradicate protoscoleces [27].

Because of the high risks of recurrence following surgery and the uncertainty of a recovery following pharmacological therapy and/or percutaneous intervention, it is now generally regarded that a close follow-up of CE patients should also be conducted for at least 5 years. Since liver toxicity and leukopenia are the most serious side effects and may limit ABZ administration in some patients, routine blood counts and serum transaminases tests (AST and ALT) are conducted to evaluate the safety of the effect within the first six months following the start of anti-helminth treatment. ABZ sulfoxide, a metabolite of ABZ, is measured to assess the patient’s adherence and to adjust the dose of medication [15].

Our study has some limitations. First, we did not perform a serologic test for *Echinococcus*. In Korea, a diagnostic panel test consisting of *Clonorchis sinensis*, *Paragonimus westermani*, cysticercosis, and *sparganum* was widely performed if the patient had any suspicions of having a parasite infection. Only when Echinococcus infection was suspected were additional tests requested. Since we initially focused on the possibility of another disease, cysticercosis, the enzyme-linked immunosorbent assay (ELISA) test for echinococcus was not performed [11]. Second, we did not consider cross-reactivity between *Cysticercus* and *Echinococcus*. There were two commonly used diagnostic tools for cysticercosis including the ELISA and enzyme-linked immunoelectrotransfer blot (EITB) [28,29]. In Korea, a GENEDIA^Ⓡ^ cysticercosis/*Sparganum* antibody test was performed using the ELISA method [30]. Due to cross-reactivity among the patients with these diseases, the low specificity of the current immunological test was posed. Since they had some phylogenetical similarities, patients with these infections could not be distinguished by ELISA due to false-positive results [31]. Nevertheless, some lessons were learned from this case. Our case presents conflicting results of radiologic and immunodiagnostic tests. An important point is that the diagnosis and management of parasitic hepatic cysts should require comprehensive approaches that are combined to accurately interpret radiologic and immunologic tests, the patient’s history taking including nationality, the histology of oversea travel, the prevalence of the parasite causing the disease, and the country where the patients lived.

## 4. Conclusions

Due to the lack of experience of hepatic parasitic infection, which was not prevalent in Korea, the clinicians had difficulty in differentiating the diseases. So, we should need to know about the etiologies that have been happening most commonly in hepatic cysts suspected of parasitic infection. Moreover, we should try to figure out the patient’s nationality, history of travel, and surrounding environment, including livestock and pets, as detail.

## Data Availability

The data supporting the findings of this study are available from the corresponding author (M-K.K.) upon reasonable request.
